# Bis[μ-2-(4-hy­droxy­phen­yl)acetato]-κ^3^ *O*,*O*′:*O*;κ^3^ *O*:*O*,*O*′-bis­{aqua­(4,4′-bipyridine-κ*N*)bis­[2-(4-hy­droxy­phen­yl)acetato-κ^2^ *O*,*O*′]dysprosium(III)} monohydrate

**DOI:** 10.1107/S1600536810044107

**Published:** 2010-11-06

**Authors:** Jia-Lu Liu, Jian-Feng Liu, Guo-Liang Zhao

**Affiliations:** a College of Chemistry and Life Sciences, Zhejiang Normal University, Jinhua 321004, People’s Republic of China; b Zhejiang Normal University Xingzhi College, Jinhua 321004, People’s Republic of China

## Abstract

In the title dinuclear complex, [Dy_2_(C_8_H_7_O_3_)_6_(C_10_H_8_N_2_)_2_(H_2_O)_2_]·H_2_O, the Dy^III^ atoms are coordinated by eight O atoms from four 2-(4-hy­droxy­phen­yl)acetate (HPAA) ligands and a water mol­ecule, and one N atom from a 4,4′-bipyridine (bipy) ligand in a distorted tricapped trigonal prismatic geometry. Whereas four HPAA ligands coordinate to just two Dy^III^ atoms, the remaining two ligands bridge the two Dy^III^ atoms. In the crystal, O—H⋯O and O—H⋯N hydrogen bonds link the mol­ecules into a three-dimensional network.

## Related literature

For background literature on metal–organic complexes, see: Fang & Zhang (2006 ▶); Wang *et al.* (2010 ▶); Wang & Sevov (2008 ▶). For our previous work, see: Liu *et al.* (2010 ▶). 
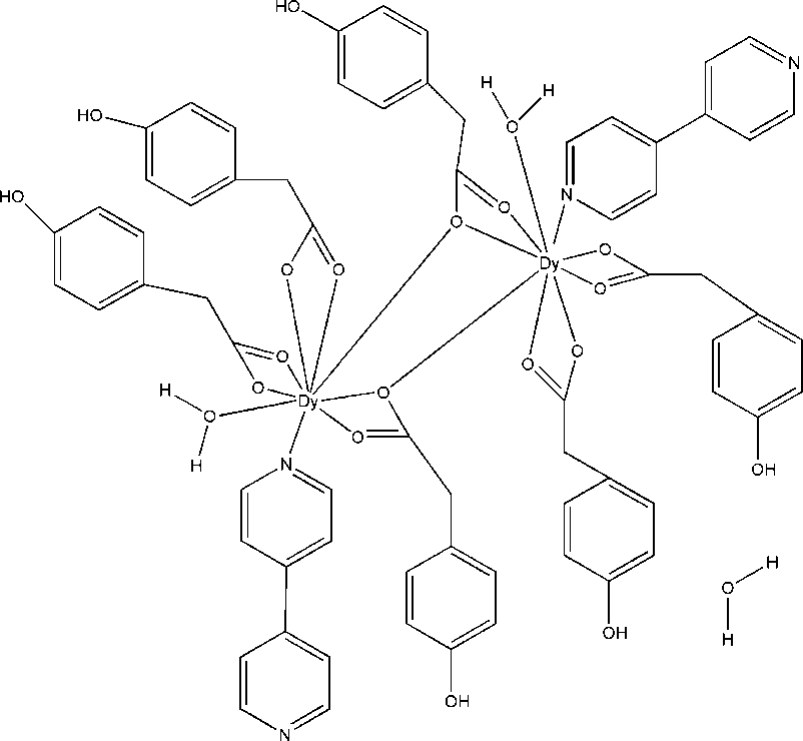


## Experimental

### 

#### Crystal data

[Dy_2_(C_8_H_7_O_3_)_6_(C_10_H_8_N_2_)_2_(H_2_O)_2_]·H_2_O
*M* *_r_* = 1598.23
Triclinic, 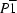

*a* = 11.7235 (1) Å
*b* = 16.2155 (2) Å
*c* = 18.4135 (2) Å
α = 83.4850 (1)°
β = 72.2430 (1)°
γ = 71.2160 (1)°
*V* = 3155.68 (6) Å^3^
*Z* = 2
Mo *K*α radiation
μ = 2.43 mm^−1^
*T* = 296 K
0.13 × 0.08 × 0.07 mm

#### Data collection

Bruker APEXII area-detector diffractometer
Absorption correction: multi-scan (*SADABS*; Sheldrick, 1996 ▶) *T*_min_ = 0.786, *T*_max_ = 0.849
40429 measured reflections
11065 independent reflections
10032 reflections with *I* > 2σ(*I*)
*R*_int_ = 0.025

#### Refinement

*R*[*F* ^2^ > 2σ(*F* ^2^)] = 0.019
*wR*(*F* ^2^) = 0.048
*S* = 1.05
11065 reflections
875 parameters
9 restraints
H atoms treated by a mixture of independent and constrained refinement
Δρ_max_ = 0.32 e Å^−3^
Δρ_min_ = −0.83 e Å^−3^

### 

Data collection: *APEX2* (Bruker, 2006 ▶); cell refinement: *SAINT* (Bruker, 2006 ▶); data reduction: *SAINT*; program(s) used to solve structure: *SHELXS97* (Sheldrick, 2008 ▶); program(s) used to refine structure: *SHELXL97* (Sheldrick, 2008 ▶); molecular graphics: *XP* (Sheldrick, 2008 ▶); software used to prepare material for publication: *SHELXTL* (Sheldrick, 2008 ▶).

## Supplementary Material

Crystal structure: contains datablocks global, I. DOI: 10.1107/S1600536810044107/bt5397sup1.cif

Structure factors: contains datablocks I. DOI: 10.1107/S1600536810044107/bt5397Isup2.hkl

Additional supplementary materials:  crystallographic information; 3D view; checkCIF report

## Figures and Tables

**Table 1:** Hydrogen-bond geometry (Å, °)

*D*—H⋯*A*	*D*—H	H⋯*A*	*D*⋯*A*	*D*—H⋯*A*
O3—H3*B*⋯O12^i^	0.82	1.93	2.742 (3)	169
O6—H6*B*⋯O3*W*^ii^	0.82	1.86	2.644 (3)	160
O9—H9*A*⋯O17^iii^	0.82	1.85	2.670 (3)	173
O12—H12*A*⋯O11^iv^	0.82	1.94	2.748 (2)	168
O15—H15*C*⋯O6^v^	0.82	1.90	2.715 (3)	174
O18—H18*B*⋯O9^ii^	0.82	1.95	2.766 (3)	174
O2*W*—H2*WA*⋯O5	0.84 (4)	1.96 (2)	2.745 (2)	154 (4)
O2*W*—H2*WB*⋯N2^ii^	0.84 (2)	2.04 (2)	2.834 (3)	160 (4)
O3*W*—H3*WB*⋯O3	0.83 (4)	1.99 (2)	2.799 (3)	163 (4)
O1*W*—H1*WA*⋯O13	0.82 (4)	1.98 (2)	2.738 (2)	153 (4)
O1*W*—H1*WB*⋯N4^i^	0.84 (2)	1.96 (2)	2.781 (3)	167 (4)
O3*W*—H3*WA*⋯O1^vi^	0.84 (4)	1.94 (2)	2.775 (3)	178 (4)

Symmetry codes: (i) 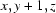; (ii) 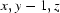; (iii) 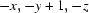; (iv) 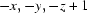; (v) 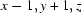; (vi) 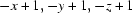.

**Fig. 1.**
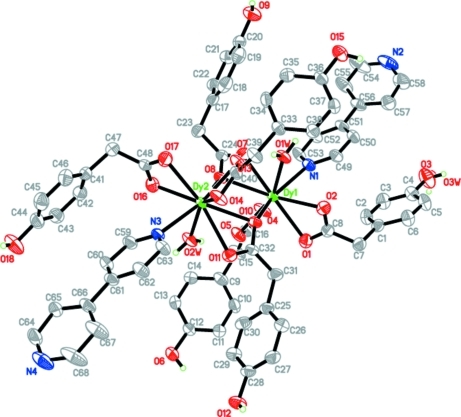
The molecular structure of the title complex, showing the atom-labelling scheme. Displacement ellipsoids are drawn at the 50% probability level.

## supplementary crystallographic information

### Comment

The design and synthesis of carboxylic mental-organic complexes have been an increasing interest for decades owing to their potential practical applications, such as fluorescence, magnetism (Wang *et al.*, 2010; Fang & Zhang, 2006; Wang *et al.*, 2008). We have worked at it before (Liu *et al.*, 2010). In the paper, we report the crystal structure of a new dysprosium ^III^ complex with the ligand *p*- hydroxyphenylacetic acid. The title compound consist of two Dy (III) cation, six *L* ligands, two bipy molecules and three water molecules. In the bicentric structure compound, every centric atom is coordinated with seven O atoms from four *L* ligands,one N atom from 4,4'-bipyridine ligand and one O atom from a water molecule. The centric atom is nine coordinated. The PAA ligands are coordinated by two modes, bridging and bridging tridentate. (Fig.1). The dysprosium ^III^ atom is in a distorted capped pentagonal prism environment. The Dy–O bond lengths range from 2.3875 (16) Å-2.5555 (16) Å. The Dy–N distances range from 2.494 (3) Å-2.509 (3) Å. The Dy–O(water) bond length range are 2.5415 (19)Å and  2.5631(19 Å. In addition, there are plenty of hydrogen bonds in the crystal structure due to the existence of dissociative water and crystal water molecules. The occurrence of numerous O–H···O involving coordinated and non- coordinated water molecules build up a three dimensional network.

### Experimental

All reagents and solvents used were of commercially available quality and without purified before using. *p*-hydroxyphenylacetic acid(HPAA) (0.456 g, 3 mmol)and sodium hydroxide (0.12 g, 3 mmol) were mixed together in water(10 ml), then Dy[(NO_3_)_3_](0.360 g, 1 mmol) dissolved in water(10 ml) was added into the above solution, after stirred for an hour, an ethanol(5 ml) solution of 4,4'-bipyridine(0.156 g, 1 mmol) is slowly dripped into the above solution with stirring for three hours. After filtration, the filtrate was allowed to stand at room temperature, and single crystals suitable for *X*-ray work were obtained after a week.

### Refinement

All H atoms attached to C atoms and *O*(hydroxyl) atom were fixed geometrically and treated as riding with C–H = 0.97 Å (methylene) or 0.93 Å (aromatic) and O–H = 0.82 Å with *U*_iso_(H) = 1.2*U*_eq_(C) or *U*_iso_(H) = 1.5*U*_eq_(O). H atoms of the water molecules were located in a difference Fourier map and included in the  refinement using restraints (O–H= 0.82 (1)Å and H···H= 1.39 (2) Å) with *U*_iso_(H) = 1.5*U*_eq_(O).

### Crystal data

**Table:**

[Dy_2_(C_8_H_7_O_3_)_6_(C_10_H_8_N_2_)_2_(H_2_O)_2_]·H_2_O	*Z* = 2
*M**_r_* = 1598.23	*F*(000) = 1600
Triclinic, *P*1	*D*_x_ = 1.682 Mg m^−^^3^
Hall symbol: -P 1	Mo *K*α radiation, λ = 0.71073 Å
*a* = 11.7235 (1) Å	Cell parameters from 9252 reflections
*b* = 16.2155 (2) Å	θ = 1.2–25.0°
*c* = 18.4135 (2) Å	µ = 2.43 mm^−^^1^
α = 83.4850 (1)°	*T* = 296 K
β = 72.2430 (1)°	Block, colourless
γ = 71.2160 (1)°	0.13 × 0.08 × 0.07 mm
*V* = 3155.68 (6) Å^3^	

### Data collection

**Table:**

Bruker APEXII area-detector diffractometer	11065 independent reflections
Radiation source: fine-focus sealed tube	10032 reflections with *I* > 2σ(*I*)
graphite	*R*_int_ = 0.025
φ and ω scans	θ_max_ = 25.0°, θ_min_ = 1.2°
Absorption correction: empirical (using intensity measurements) (*SADABS*; Sheldrick, 1996)	*h* = −13→13
*T*_min_ = 0.786, *T*_max_ = 0.849	*k* = −19→19
40429 measured reflections	*l* = −21→21

### Refinement

**Table:**

Refinement on *F*^2^	Primary atom site location: structure-invariant direct methods
Least-squares matrix: full	Secondary atom site location: difference Fourier map
*R*[*F*^2^ > 2σ(*F*^2^)] = 0.019	Hydrogen site location: inferred from neighbouring sites
*wR*(*F*^2^) = 0.048	H atoms treated by a mixture of independent and constrained refinement
*S* = 1.05	*w* = 1/[σ^2^(*F*_o_^2^) + (0.0215*P*)^2^ + 1.845*P*] where *P* = (*F*_o_^2^ + 2*F*_c_^2^)/3
11065 reflections	(Δ/σ)_max_ = 0.002
875 parameters	Δρ_max_ = 0.32 e Å^−^^3^
9 restraints	Δρ_min_ = −0.83 e Å^−^^3^

### Special details

**Table:**

Geometry. All e.s.d.'s (except the e.s.d. in the dihedral angle between two l.s. planes) are estimated using the full covariance matrix. The cell e.s.d.'s are taken into account individually in the estimation of e.s.d.'s in distances, angles and torsion angles; correlations between e.s.d.'s in cell parameters are only used when they are defined by crystal symmetry. An approximate (isotropic) treatment of cell e.s.d.'s is used for estimating e.s.d.'s involving l.s. planes.
Refinement. Refinement of *F*^2^ against ALL reflections. The weighted *R*-factor *wR* and goodness of fit *S* are based on *F*^2^, conventional *R*-factors *R* are based on *F*, with *F* set to zero for negative *F*^2^. The threshold expression of *F*^2^ > σ(*F*^2^) is used only for calculating *R*-factors(gt) *etc*. and is not relevant to the choice of reflections for refinement. *R*-factors based on *F*^2^ are statistically about twice as large as those based on *F*, and *R*- factors based on ALL data will be even larger.

### Fractional atomic coordinates and isotropic or equivalent isotropic displacement parameters (Å^2^)

**Table:**

	*x*	*y*	*z*	*U*_iso_*/*U*_eq_	
Dy1	0.271981 (9)	0.363948 (6)	0.282840 (6)	0.02273 (4)	
Dy2	0.131125 (9)	0.207461 (6)	0.197161 (6)	0.02254 (4)	
N1	0.31242 (19)	0.50888 (12)	0.28243 (12)	0.0309 (4)	
N2	0.3936 (3)	0.92825 (16)	0.2213 (2)	0.0619 (8)	
N3	0.08633 (19)	0.06439 (12)	0.19011 (11)	0.0298 (4)	
N4	0.0329 (3)	−0.36289 (16)	0.2296 (2)	0.0627 (8)	
C1	0.2255 (2)	0.50889 (16)	0.51605 (14)	0.0327 (5)	
C2	0.3410 (2)	0.51461 (18)	0.47221 (15)	0.0413 (6)	
H2A	0.3971	0.4675	0.4427	0.050*	
C3	0.3756 (3)	0.58875 (19)	0.47098 (16)	0.0446 (7)	
H3A	0.4540	0.5913	0.4406	0.053*	
C4	0.2936 (2)	0.65878 (17)	0.51481 (16)	0.0397 (6)	
C5	0.1790 (3)	0.65370 (18)	0.56058 (17)	0.0441 (7)	
H5A	0.1244	0.7001	0.5915	0.053*	
C6	0.1448 (2)	0.57973 (17)	0.56079 (16)	0.0408 (6)	
H6A	0.0664	0.5773	0.5914	0.049*	
C7	0.1860 (3)	0.42785 (17)	0.51933 (14)	0.0387 (6)	
H7A	0.2312	0.3825	0.5484	0.046*	
H7B	0.0969	0.4409	0.5452	0.046*	
C8	0.2127 (2)	0.39551 (16)	0.44026 (14)	0.0326 (6)	
C9	0.6224 (2)	0.08968 (16)	0.30225 (16)	0.0357 (6)	
C10	0.6039 (3)	0.05837 (17)	0.37600 (17)	0.0463 (7)	
H10A	0.5913	0.0955	0.4146	0.056*	
C11	0.6034 (3)	−0.02653 (17)	0.39483 (16)	0.0464 (7)	
H11A	0.5914	−0.0462	0.4452	0.056*	
C12	0.6207 (2)	−0.08148 (16)	0.33835 (15)	0.0356 (6)	
C13	0.6383 (3)	−0.05151 (17)	0.26432 (15)	0.0422 (7)	
H13A	0.6500	−0.0886	0.2259	0.051*	
C14	0.6389 (3)	0.03275 (17)	0.24648 (16)	0.0406 (6)	
H14A	0.6506	0.0521	0.1960	0.049*	
C15	0.6248 (3)	0.18147 (16)	0.28169 (19)	0.0463 (7)	
H15A	0.6908	0.1806	0.2344	0.056*	
H15B	0.6471	0.2027	0.3210	0.056*	
C16	0.5045 (2)	0.24494 (15)	0.27220 (14)	0.0306 (5)	
C17	0.2727 (3)	0.45387 (17)	0.01337 (14)	0.0392 (6)	
C18	0.1464 (3)	0.50122 (19)	0.02819 (15)	0.0450 (7)	
H18A	0.0859	0.4745	0.0544	0.054*	
C19	0.1087 (3)	0.58734 (19)	0.00474 (15)	0.0428 (7)	
H19A	0.0237	0.6181	0.0153	0.051*	
C20	0.1978 (3)	0.62747 (16)	−0.03442 (15)	0.0357 (6)	
C21	0.3236 (2)	0.58150 (16)	−0.04913 (15)	0.0379 (6)	
H21A	0.3840	0.6084	−0.0750	0.046*	
C22	0.3600 (3)	0.49590 (17)	−0.02560 (15)	0.0406 (6)	
H22A	0.4452	0.4656	−0.0361	0.049*	
C23	0.3125 (4)	0.36042 (18)	0.04013 (16)	0.0534 (8)	
H23A	0.2577	0.3307	0.0322	0.064*	
H23B	0.3975	0.3317	0.0099	0.064*	
C24	0.3078 (2)	0.35337 (15)	0.12322 (14)	0.0306 (5)	
C25	0.1422 (3)	0.11408 (16)	0.47622 (14)	0.0358 (6)	
C26	0.0395 (3)	0.10685 (17)	0.53601 (15)	0.0395 (6)	
H26A	−0.0292	0.1560	0.5518	0.047*	
C27	0.0380 (3)	0.02737 (17)	0.57250 (15)	0.0393 (6)	
H27A	−0.0309	0.0235	0.6128	0.047*	
C28	0.1386 (2)	−0.04553 (16)	0.54891 (14)	0.0328 (6)	
C29	0.2421 (3)	−0.03927 (17)	0.48987 (15)	0.0390 (6)	
H29A	0.3108	−0.0885	0.4742	0.047*	
C30	0.2431 (3)	0.04010 (18)	0.45449 (15)	0.0401 (6)	
H30A	0.3132	0.0439	0.4151	0.048*	
C31	0.1441 (3)	0.19920 (17)	0.43400 (15)	0.0425 (7)	
H31A	0.0804	0.2462	0.4653	0.051*	
H31B	0.2251	0.2074	0.4274	0.051*	
C32	0.1217 (2)	0.20597 (14)	0.35722 (13)	0.0275 (5)	
C33	−0.2543 (2)	0.47975 (15)	0.24487 (14)	0.0286 (5)	
C34	−0.2115 (2)	0.51805 (16)	0.17475 (14)	0.0348 (6)	
H34A	−0.1733	0.4836	0.1317	0.042*	
C35	−0.2243 (2)	0.60543 (17)	0.16740 (15)	0.0376 (6)	
H35A	−0.1955	0.6296	0.1197	0.045*	
C36	−0.2801 (2)	0.65755 (15)	0.23086 (15)	0.0334 (6)	
C37	−0.3226 (2)	0.62099 (16)	0.30114 (15)	0.0371 (6)	
H37A	−0.3602	0.6556	0.3442	0.045*	
C38	−0.3092 (2)	0.53266 (16)	0.30763 (14)	0.0350 (6)	
H38A	−0.3378	0.5085	0.3553	0.042*	
C39	−0.2412 (2)	0.38403 (15)	0.25171 (16)	0.0347 (6)	
H39A	−0.2861	0.3716	0.3033	0.042*	
H39B	−0.2819	0.3712	0.2176	0.042*	
C40	−0.1089 (2)	0.32384 (14)	0.23409 (13)	0.0279 (5)	
C41	0.2338 (3)	0.05507 (16)	−0.04528 (14)	0.0367 (6)	
C42	0.3527 (3)	0.00417 (17)	−0.04189 (14)	0.0388 (6)	
H42A	0.4092	0.0313	−0.0376	0.047*	
C43	0.3893 (3)	−0.08532 (17)	−0.04468 (15)	0.0424 (6)	
H43A	0.4698	−0.1179	−0.0429	0.051*	
C44	0.3062 (3)	−0.12652 (17)	−0.05013 (16)	0.0436 (7)	
C45	0.1881 (3)	−0.07749 (19)	−0.05483 (16)	0.0466 (7)	
H45A	0.1321	−0.1049	−0.0593	0.056*	
C46	0.1531 (3)	0.01245 (18)	−0.05282 (15)	0.0428 (6)	
H46A	0.0737	0.0449	−0.0566	0.051*	
C47	0.1913 (3)	0.15330 (17)	−0.03899 (15)	0.0429 (7)	
H47A	0.2545	0.1764	−0.0743	0.051*	
H47B	0.1138	0.1776	−0.0532	0.051*	
C48	0.1703 (3)	0.18082 (15)	0.04088 (14)	0.0332 (6)	
C49	0.2297 (2)	0.57803 (15)	0.32198 (15)	0.0337 (6)	
H49A	0.1564	0.5712	0.3563	0.040*	
C50	0.2471 (2)	0.65933 (16)	0.31464 (15)	0.0349 (6)	
H50A	0.1869	0.7054	0.3439	0.042*	
C51	0.3547 (2)	0.67157 (15)	0.26344 (15)	0.0337 (6)	
C52	0.4424 (2)	0.59920 (16)	0.22376 (17)	0.0398 (6)	
H52A	0.5173	0.6039	0.1899	0.048*	
C53	0.4181 (2)	0.52075 (16)	0.23468 (16)	0.0373 (6)	
H53A	0.4782	0.4732	0.2075	0.045*	
C54	0.4487 (3)	0.8668 (2)	0.1689 (2)	0.0698 (11)	
H54A	0.4954	0.8814	0.1214	0.084*	
C55	0.4415 (3)	0.78259 (18)	0.1802 (2)	0.0569 (9)	
H55A	0.4820	0.7424	0.1412	0.068*	
C56	0.3732 (2)	0.75912 (16)	0.25023 (17)	0.0391 (6)	
C57	0.3181 (3)	0.82196 (19)	0.30564 (19)	0.0584 (9)	
H57A	0.2731	0.8091	0.3543	0.070*	
C58	0.3309 (4)	0.9042 (2)	0.2879 (2)	0.0685 (10)	
H58A	0.2922	0.9457	0.3259	0.082*	
C59	0.1726 (2)	−0.00161 (15)	0.14834 (15)	0.0359 (6)	
H59A	0.2459	0.0078	0.1163	0.043*	
C60	0.1586 (3)	−0.08352 (16)	0.15017 (15)	0.0396 (6)	
H60A	0.2210	−0.1270	0.1191	0.047*	
C61	0.0521 (2)	−0.10088 (15)	0.19800 (15)	0.0346 (6)	
C62	−0.0397 (3)	−0.03106 (16)	0.23919 (16)	0.0394 (6)	
H62A	−0.1148	−0.0383	0.2707	0.047*	
C63	−0.0198 (2)	0.04895 (16)	0.23348 (15)	0.0358 (6)	
H63A	−0.0834	0.0947	0.2612	0.043*	
C64	0.1127 (4)	−0.3394 (2)	0.1692 (2)	0.0668 (11)	
H64A	0.1675	−0.3821	0.1345	0.080*	
C65	0.1195 (4)	−0.25522 (18)	0.15470 (18)	0.0581 (9)	
H65A	0.1759	−0.2421	0.1106	0.070*	
C66	0.0414 (3)	−0.19047 (16)	0.20662 (17)	0.0408 (7)	
C67	−0.0427 (3)	−0.21453 (19)	0.2695 (2)	0.0572 (9)	
H67A	−0.0977	−0.1736	0.3058	0.069*	
C68	−0.0440 (3)	−0.3001 (2)	0.2780 (3)	0.0688 (11)	
H68A	−0.1024	−0.3147	0.3201	0.083*	
O1W	0.08706 (17)	0.45800 (11)	0.25422 (11)	0.0358 (4)	
O1	0.31122 (18)	0.33502 (12)	0.41166 (10)	0.0428 (4)	
O2W	0.32368 (16)	0.11269 (11)	0.21589 (12)	0.0370 (4)	
O2	0.13729 (16)	0.43112 (11)	0.40150 (10)	0.0363 (4)	
O3	0.3302 (2)	0.73175 (14)	0.51198 (14)	0.0615 (6)	
H3B	0.2720	0.7699	0.5379	0.061 (11)*	
O3W	0.5567 (2)	0.76698 (14)	0.49124 (13)	0.0546 (5)	
O4	0.49442 (16)	0.32416 (11)	0.26346 (12)	0.0428 (5)	
O5	0.41195 (15)	0.21883 (10)	0.27596 (11)	0.0363 (4)	
O6	0.6236 (2)	−0.16704 (12)	0.35244 (11)	0.0555 (6)	
H6B	0.5966	−0.1750	0.3984	0.083*	
O7	0.35201 (18)	0.39913 (12)	0.14945 (10)	0.0424 (5)	
O8	0.25644 (15)	0.30037 (10)	0.16668 (9)	0.0288 (4)	
O9	0.16591 (19)	0.71256 (12)	−0.05939 (12)	0.0521 (5)	
H9A	0.0951	0.7267	−0.0646	0.078*	
O10	0.14384 (15)	0.26908 (10)	0.31314 (9)	0.0278 (3)	
O11	0.08268 (17)	0.15277 (11)	0.33518 (10)	0.0347 (4)	
O12	0.14160 (18)	−0.12619 (11)	0.58221 (11)	0.0429 (4)	
H12A	0.0698	−0.1267	0.6050	0.064*	
O13	−0.01518 (15)	0.35165 (10)	0.20656 (11)	0.0352 (4)	
O14	−0.09143 (16)	0.24329 (10)	0.24589 (11)	0.0392 (4)	
O15	−0.2902 (2)	0.74396 (11)	0.22045 (12)	0.0506 (5)	
H15C	−0.3167	0.7674	0.2620	0.076*	
O16	0.25855 (16)	0.15612 (11)	0.07111 (10)	0.0369 (4)	
O17	0.06366 (18)	0.22777 (12)	0.07857 (10)	0.0445 (5)	
O18	0.3458 (2)	−0.21560 (13)	−0.05106 (15)	0.0680 (7)	
H18B	0.2894	−0.2331	−0.0545	0.102*	
H2WA	0.372 (3)	0.131 (2)	0.231 (2)	0.102*	
H2WB	0.362 (3)	0.0595 (12)	0.211 (2)	0.102*	
H3WB	0.499 (3)	0.746 (2)	0.495 (2)	0.102*	
H1WA	0.036 (3)	0.441 (2)	0.243 (2)	0.102*	
H1WB	0.059 (4)	0.5121 (11)	0.250 (2)	0.102*	
H3WA	0.595 (3)	0.736 (2)	0.521 (2)	0.102*	

### Atomic displacement parameters (Å^2^)

**Table:**

	*U*^11^	*U*^22^	*U*^33^	*U*^12^	*U*^13^	*U*^23^
Dy1	0.02541 (6)	0.01619 (6)	0.02990 (6)	−0.00870 (4)	−0.01104 (5)	0.00238 (4)
Dy2	0.02400 (6)	0.01511 (6)	0.03053 (6)	−0.00689 (4)	−0.00989 (5)	0.00033 (4)
N1	0.0316 (11)	0.0229 (10)	0.0412 (12)	−0.0107 (9)	−0.0127 (9)	0.0007 (9)
N2	0.0572 (17)	0.0303 (14)	0.113 (3)	−0.0220 (13)	−0.0433 (17)	0.0180 (15)
N3	0.0358 (11)	0.0207 (10)	0.0347 (11)	−0.0091 (9)	−0.0125 (9)	0.0008 (8)
N4	0.080 (2)	0.0295 (14)	0.109 (3)	−0.0250 (14)	−0.0667 (19)	0.0163 (15)
C1	0.0360 (14)	0.0354 (14)	0.0297 (13)	−0.0123 (11)	−0.0120 (11)	−0.0007 (10)
C2	0.0363 (15)	0.0406 (15)	0.0425 (15)	−0.0079 (12)	−0.0051 (12)	−0.0116 (12)
C3	0.0315 (14)	0.0498 (17)	0.0499 (17)	−0.0167 (13)	−0.0014 (12)	−0.0066 (13)
C4	0.0352 (14)	0.0378 (15)	0.0501 (16)	−0.0137 (12)	−0.0147 (12)	−0.0023 (12)
C5	0.0361 (15)	0.0361 (15)	0.0560 (18)	−0.0064 (12)	−0.0075 (13)	−0.0132 (13)
C6	0.0315 (14)	0.0418 (15)	0.0473 (16)	−0.0136 (12)	−0.0034 (12)	−0.0077 (12)
C7	0.0495 (16)	0.0371 (14)	0.0320 (14)	−0.0186 (13)	−0.0098 (12)	0.0003 (11)
C8	0.0418 (15)	0.0311 (13)	0.0313 (13)	−0.0224 (12)	−0.0086 (11)	0.0024 (11)
C9	0.0241 (12)	0.0268 (13)	0.0572 (17)	−0.0033 (10)	−0.0179 (12)	0.0005 (12)
C10	0.0626 (19)	0.0301 (14)	0.0484 (17)	−0.0058 (13)	−0.0245 (15)	−0.0098 (12)
C11	0.066 (2)	0.0342 (15)	0.0357 (15)	−0.0094 (14)	−0.0166 (14)	−0.0007 (12)
C12	0.0398 (15)	0.0257 (13)	0.0403 (15)	−0.0082 (11)	−0.0117 (12)	−0.0011 (11)
C13	0.0581 (18)	0.0318 (14)	0.0360 (15)	−0.0127 (13)	−0.0115 (13)	−0.0064 (11)
C14	0.0476 (16)	0.0343 (14)	0.0378 (15)	−0.0111 (12)	−0.0123 (12)	0.0038 (11)
C15	0.0361 (15)	0.0270 (14)	0.081 (2)	−0.0085 (12)	−0.0280 (15)	0.0048 (13)
C16	0.0317 (13)	0.0250 (13)	0.0371 (14)	−0.0096 (11)	−0.0121 (11)	0.0011 (10)
C17	0.0662 (19)	0.0366 (14)	0.0268 (13)	−0.0283 (14)	−0.0182 (13)	0.0029 (11)
C18	0.0596 (19)	0.0584 (19)	0.0303 (14)	−0.0405 (16)	−0.0086 (13)	0.0025 (13)
C19	0.0410 (16)	0.0537 (18)	0.0385 (15)	−0.0190 (14)	−0.0113 (12)	−0.0067 (13)
C20	0.0465 (16)	0.0329 (14)	0.0376 (14)	−0.0170 (12)	−0.0210 (12)	0.0004 (11)
C21	0.0404 (15)	0.0343 (14)	0.0466 (16)	−0.0203 (12)	−0.0161 (12)	0.0065 (12)
C22	0.0466 (16)	0.0376 (15)	0.0442 (16)	−0.0166 (13)	−0.0204 (13)	0.0047 (12)
C23	0.100 (3)	0.0373 (16)	0.0362 (16)	−0.0348 (17)	−0.0258 (16)	0.0069 (12)
C24	0.0380 (14)	0.0241 (12)	0.0311 (13)	−0.0124 (11)	−0.0101 (11)	0.0032 (10)
C25	0.0525 (16)	0.0334 (14)	0.0334 (14)	−0.0230 (13)	−0.0216 (12)	0.0079 (11)
C26	0.0452 (15)	0.0328 (14)	0.0393 (15)	−0.0098 (12)	−0.0134 (12)	0.0013 (11)
C27	0.0452 (16)	0.0400 (15)	0.0330 (14)	−0.0185 (13)	−0.0080 (12)	0.0058 (11)
C28	0.0406 (14)	0.0315 (13)	0.0344 (14)	−0.0185 (12)	−0.0175 (11)	0.0091 (10)
C29	0.0387 (15)	0.0363 (14)	0.0409 (15)	−0.0117 (12)	−0.0112 (12)	0.0046 (12)
C30	0.0424 (15)	0.0472 (16)	0.0357 (14)	−0.0244 (13)	−0.0106 (12)	0.0101 (12)
C31	0.0684 (19)	0.0347 (14)	0.0409 (15)	−0.0296 (14)	−0.0280 (14)	0.0104 (12)
C32	0.0281 (12)	0.0219 (12)	0.0317 (13)	−0.0084 (10)	−0.0082 (10)	0.0039 (10)
C33	0.0215 (12)	0.0238 (12)	0.0395 (14)	−0.0030 (10)	−0.0102 (10)	−0.0033 (10)
C34	0.0367 (14)	0.0305 (13)	0.0336 (14)	−0.0049 (11)	−0.0067 (11)	−0.0105 (11)
C35	0.0440 (15)	0.0349 (14)	0.0330 (14)	−0.0137 (12)	−0.0090 (12)	0.0028 (11)
C36	0.0353 (14)	0.0237 (12)	0.0446 (15)	−0.0074 (11)	−0.0174 (12)	−0.0019 (11)
C37	0.0436 (15)	0.0303 (13)	0.0346 (14)	−0.0045 (12)	−0.0112 (12)	−0.0095 (11)
C38	0.0362 (14)	0.0309 (13)	0.0320 (13)	−0.0036 (11)	−0.0079 (11)	−0.0012 (10)
C39	0.0282 (13)	0.0245 (12)	0.0505 (16)	−0.0069 (10)	−0.0105 (11)	−0.0021 (11)
C40	0.0298 (13)	0.0229 (12)	0.0328 (13)	−0.0067 (10)	−0.0113 (10)	−0.0038 (10)
C41	0.0508 (16)	0.0336 (14)	0.0250 (13)	−0.0145 (12)	−0.0079 (11)	−0.0012 (10)
C42	0.0438 (16)	0.0403 (15)	0.0358 (14)	−0.0203 (13)	−0.0086 (12)	0.0000 (11)
C43	0.0429 (16)	0.0384 (15)	0.0438 (16)	−0.0108 (13)	−0.0109 (12)	−0.0010 (12)
C44	0.0531 (18)	0.0349 (15)	0.0426 (16)	−0.0171 (13)	−0.0085 (13)	−0.0033 (12)
C45	0.0504 (18)	0.0497 (17)	0.0468 (17)	−0.0268 (15)	−0.0096 (13)	−0.0060 (13)
C46	0.0411 (15)	0.0460 (16)	0.0404 (15)	−0.0110 (13)	−0.0104 (12)	−0.0069 (12)
C47	0.0585 (18)	0.0351 (15)	0.0321 (14)	−0.0100 (13)	−0.0132 (13)	−0.0002 (11)
C48	0.0498 (16)	0.0201 (12)	0.0311 (13)	−0.0137 (12)	−0.0118 (12)	0.0037 (10)
C49	0.0341 (14)	0.0262 (13)	0.0423 (15)	−0.0123 (11)	−0.0106 (11)	0.0023 (11)
C50	0.0378 (14)	0.0241 (12)	0.0437 (15)	−0.0085 (11)	−0.0131 (12)	−0.0028 (11)
C51	0.0375 (14)	0.0220 (12)	0.0494 (15)	−0.0111 (11)	−0.0228 (12)	0.0039 (11)
C52	0.0285 (13)	0.0287 (14)	0.0617 (18)	−0.0124 (11)	−0.0095 (12)	0.0031 (12)
C53	0.0312 (14)	0.0229 (13)	0.0561 (17)	−0.0082 (11)	−0.0089 (12)	−0.0036 (11)
C54	0.0450 (19)	0.0388 (18)	0.116 (3)	−0.0196 (15)	−0.0111 (19)	0.023 (2)
C55	0.0424 (17)	0.0312 (15)	0.085 (2)	−0.0126 (13)	−0.0014 (16)	0.0041 (15)
C56	0.0383 (15)	0.0259 (13)	0.0629 (18)	−0.0146 (12)	−0.0260 (13)	0.0072 (12)
C57	0.095 (3)	0.0369 (16)	0.058 (2)	−0.0314 (17)	−0.0324 (18)	0.0020 (14)
C58	0.107 (3)	0.0357 (17)	0.087 (3)	−0.0317 (19)	−0.052 (2)	0.0004 (17)
C59	0.0408 (15)	0.0258 (13)	0.0389 (15)	−0.0121 (11)	−0.0075 (12)	0.0027 (11)
C60	0.0511 (17)	0.0236 (13)	0.0429 (15)	−0.0077 (12)	−0.0149 (13)	−0.0028 (11)
C61	0.0461 (15)	0.0247 (12)	0.0449 (15)	−0.0149 (11)	−0.0279 (13)	0.0059 (11)
C62	0.0369 (14)	0.0303 (14)	0.0545 (17)	−0.0153 (12)	−0.0135 (13)	0.0019 (12)
C63	0.0359 (14)	0.0229 (12)	0.0501 (16)	−0.0105 (11)	−0.0121 (12)	−0.0024 (11)
C64	0.123 (3)	0.0313 (16)	0.071 (2)	−0.0256 (19)	−0.061 (2)	0.0007 (15)
C65	0.109 (3)	0.0319 (15)	0.0502 (18)	−0.0292 (17)	−0.0403 (19)	0.0036 (13)
C66	0.0529 (17)	0.0241 (13)	0.0626 (18)	−0.0169 (12)	−0.0380 (15)	0.0066 (12)
C67	0.0402 (17)	0.0330 (16)	0.102 (3)	−0.0161 (13)	−0.0255 (17)	0.0126 (16)
C68	0.051 (2)	0.0351 (18)	0.131 (3)	−0.0234 (16)	−0.040 (2)	0.026 (2)
O1W	0.0391 (10)	0.0197 (8)	0.0547 (11)	−0.0077 (8)	−0.0242 (9)	0.0012 (8)
O1	0.0491 (12)	0.0382 (10)	0.0401 (10)	−0.0050 (9)	−0.0184 (9)	−0.0056 (8)
O2W	0.0348 (10)	0.0197 (8)	0.0618 (12)	−0.0053 (8)	−0.0242 (9)	−0.0009 (8)
O2	0.0367 (10)	0.0388 (10)	0.0366 (10)	−0.0148 (8)	−0.0117 (8)	−0.0001 (8)
O3	0.0478 (13)	0.0442 (12)	0.0935 (17)	−0.0235 (11)	−0.0068 (12)	−0.0142 (12)
O3W	0.0581 (14)	0.0522 (13)	0.0582 (14)	−0.0221 (11)	−0.0242 (11)	0.0174 (10)
O4	0.0319 (10)	0.0231 (9)	0.0745 (14)	−0.0104 (8)	−0.0184 (9)	0.0097 (9)
O5	0.0305 (9)	0.0226 (8)	0.0628 (12)	−0.0070 (7)	−0.0236 (8)	−0.0032 (8)
O6	0.0925 (17)	0.0303 (10)	0.0436 (12)	−0.0255 (11)	−0.0118 (11)	−0.0006 (8)
O7	0.0604 (12)	0.0465 (11)	0.0356 (10)	−0.0384 (10)	−0.0140 (9)	0.0052 (8)
O8	0.0320 (9)	0.0209 (8)	0.0345 (9)	−0.0122 (7)	−0.0081 (7)	0.0036 (7)
O9	0.0557 (13)	0.0344 (11)	0.0765 (15)	−0.0148 (9)	−0.0360 (11)	0.0091 (10)
O10	0.0315 (9)	0.0218 (8)	0.0327 (9)	−0.0134 (7)	−0.0095 (7)	0.0058 (7)
O11	0.0475 (11)	0.0329 (9)	0.0352 (10)	−0.0261 (8)	−0.0163 (8)	0.0074 (7)
O12	0.0459 (11)	0.0328 (10)	0.0499 (11)	−0.0183 (9)	−0.0109 (9)	0.0112 (8)
O13	0.0264 (9)	0.0215 (8)	0.0601 (12)	−0.0075 (7)	−0.0148 (8)	−0.0033 (8)
O14	0.0302 (9)	0.0203 (9)	0.0609 (12)	−0.0057 (7)	−0.0076 (8)	0.0021 (8)
O15	0.0744 (14)	0.0247 (9)	0.0566 (13)	−0.0160 (9)	−0.0232 (11)	−0.0013 (8)
O16	0.0381 (10)	0.0348 (10)	0.0402 (10)	−0.0130 (8)	−0.0111 (8)	−0.0046 (8)
O17	0.0467 (11)	0.0412 (11)	0.0386 (10)	0.0034 (9)	−0.0179 (9)	−0.0070 (8)
O18	0.0727 (16)	0.0322 (11)	0.1045 (19)	−0.0179 (11)	−0.0302 (14)	−0.0027 (11)

### Geometric parameters (Å, °)

**Table:**

Dy1—O1W	2.3851 (16)	C28—O12	1.375 (3)
Dy1—O5	2.3875 (16)	C28—C29	1.384 (4)
Dy1—O10	2.3910 (15)	C29—C30	1.377 (4)
Dy1—O4	2.3970 (17)	C29—H29A	0.9300
Dy1—O2	2.4045 (17)	C30—H30A	0.9300
Dy1—O7	2.4207 (17)	C31—C32	1.501 (3)
Dy1—O1	2.5163 (18)	C31—H31A	0.9700
Dy1—N1	2.5415 (19)	C31—H31B	0.9700
Dy1—O8	2.5555 (16)	C32—O11	1.251 (3)
Dy1—C16	2.750 (2)	C32—O10	1.276 (3)
Dy1—C8	2.833 (2)	C33—C38	1.379 (3)
Dy1—C24	2.857 (2)	C33—C34	1.388 (3)
Dy2—O8	2.3395 (15)	C33—C39	1.504 (3)
Dy2—O14	2.3800 (17)	C34—C35	1.371 (3)
Dy2—O2W	2.3868 (16)	C34—H34A	0.9300
Dy2—O13	2.4013 (16)	C35—C36	1.382 (4)
Dy2—O16	2.4155 (17)	C35—H35A	0.9300
Dy2—O17	2.4950 (18)	C36—O15	1.364 (3)
Dy2—O10	2.5210 (16)	C36—C37	1.377 (4)
Dy2—O11	2.5495 (16)	C37—C38	1.385 (3)
Dy2—N3	2.5631 (19)	C37—H37A	0.9300
Dy2—C40	2.766 (2)	C38—H38A	0.9300
Dy2—C48	2.833 (2)	C39—C40	1.501 (3)
Dy2—C32	2.913 (2)	C39—H39A	0.9700
N1—C49	1.335 (3)	C39—H39B	0.9700
N1—C53	1.340 (3)	C40—O14	1.259 (3)
N2—C58	1.312 (5)	C40—O13	1.263 (3)
N2—C54	1.328 (5)	C41—C46	1.384 (4)
N3—C59	1.332 (3)	C41—C42	1.389 (4)
N3—C63	1.341 (3)	C41—C47	1.515 (4)
N4—C64	1.325 (5)	C42—C43	1.377 (4)
N4—C68	1.329 (5)	C42—H42A	0.9300
C1—C2	1.375 (4)	C43—C44	1.378 (4)
C1—C6	1.390 (4)	C43—H43A	0.9300
C1—C7	1.517 (3)	C44—O18	1.368 (3)
C2—C3	1.383 (4)	C44—C45	1.380 (4)
C2—H2A	0.9300	C45—C46	1.384 (4)
C3—C4	1.377 (4)	C45—H45A	0.9300
C3—H3A	0.9300	C46—H46A	0.9300
C4—O3	1.373 (3)	C47—C48	1.510 (3)
C4—C5	1.374 (4)	C47—H47A	0.9700
C5—C6	1.381 (4)	C47—H47B	0.9700
C5—H5A	0.9300	C48—O16	1.255 (3)
C6—H6A	0.9300	C48—O17	1.266 (3)
C7—C8	1.511 (3)	C49—C50	1.383 (3)
C7—H7A	0.9700	C49—H49A	0.9300
C7—H7B	0.9700	C50—C51	1.384 (4)
C8—O2	1.260 (3)	C50—H50A	0.9300
C8—O1	1.261 (3)	C51—C52	1.388 (4)
C9—C10	1.373 (4)	C51—C56	1.486 (3)
C9—C14	1.387 (4)	C52—C53	1.371 (3)
C9—C15	1.501 (3)	C52—H52A	0.9300
C10—C11	1.383 (4)	C53—H53A	0.9300
C10—H10A	0.9300	C54—C55	1.384 (4)
C11—C12	1.372 (4)	C54—H54A	0.9300
C11—H11A	0.9300	C55—C56	1.379 (4)
C12—C13	1.371 (4)	C55—H55A	0.9300
C12—O6	1.373 (3)	C56—C57	1.381 (4)
C13—C14	1.370 (4)	C57—C58	1.382 (4)
C13—H13A	0.9300	C57—H57A	0.9300
C14—H14A	0.9300	C58—H58A	0.9300
C15—C16	1.502 (3)	C59—C60	1.385 (3)
C15—H15A	0.9700	C59—H59A	0.9300
C15—H15B	0.9700	C60—C61	1.383 (4)
C16—O4	1.248 (3)	C60—H60A	0.9300
C16—O5	1.267 (3)	C61—C62	1.388 (4)
C17—C22	1.384 (4)	C61—C66	1.485 (3)
C17—C18	1.388 (4)	C62—C63	1.378 (3)
C17—C23	1.507 (4)	C62—H62A	0.9300
C18—C19	1.384 (4)	C63—H63A	0.9300
C18—H18A	0.9300	C64—C65	1.385 (4)
C19—C20	1.381 (4)	C64—H64A	0.9300
C19—H19A	0.9300	C65—C66	1.389 (4)
C20—O9	1.372 (3)	C65—H65A	0.9300
C20—C21	1.377 (4)	C66—C67	1.384 (4)
C21—C22	1.375 (4)	C67—C68	1.384 (4)
C21—H21A	0.9300	C67—H67A	0.9300
C22—H22A	0.9300	C68—H68A	0.9300
C23—C24	1.506 (3)	O1W—H1WA	0.82 (4)
C23—H23A	0.9700	O1W—H1WB	0.836 (18)
C23—H23B	0.9700	O2W—H2WA	0.84 (4)
C24—O7	1.239 (3)	O2W—H2WB	0.835 (18)
C24—O8	1.274 (3)	O3—H3B	0.8200
C25—C30	1.380 (4)	O3W—H3WB	0.83 (4)
C25—C26	1.389 (4)	O3W—H3WA	0.84 (4)
C25—C31	1.509 (3)	O6—H6B	0.8200
C26—C27	1.387 (4)	O9—H9A	0.8200
C26—H26A	0.9300	O12—H12A	0.8200
C27—C28	1.372 (4)	O15—H15C	0.8200
C27—H27A	0.9300	O18—H18B	0.8200
			
O1W—Dy1—O5	145.49 (6)	C17—C18—H18A	119.3
O1W—Dy1—O10	79.76 (6)	C20—C19—C18	119.8 (3)
O5—Dy1—O10	73.51 (5)	C20—C19—H19A	120.1
O1W—Dy1—O4	149.48 (6)	C18—C19—H19A	120.1
O5—Dy1—O4	54.16 (5)	O9—C20—C21	118.1 (2)
O10—Dy1—O4	127.33 (5)	O9—C20—C19	122.3 (2)
O1W—Dy1—O2	74.64 (6)	C21—C20—C19	119.6 (2)
O5—Dy1—O2	122.73 (6)	C20—C21—C22	120.2 (2)
O10—Dy1—O2	84.12 (6)	C20—C21—H21A	119.9
O4—Dy1—O2	117.40 (6)	C22—C21—H21A	119.9
O1W—Dy1—O7	77.93 (7)	C21—C22—C17	121.5 (3)
O5—Dy1—O7	95.15 (7)	C21—C22—H22A	119.2
O10—Dy1—O7	117.23 (5)	C17—C22—H22A	119.2
O4—Dy1—O7	76.64 (7)	C24—C23—C17	112.0 (2)
O2—Dy1—O7	141.29 (6)	C24—C23—H23A	109.2
O1W—Dy1—O1	127.01 (6)	C17—C23—H23A	109.2
O5—Dy1—O1	75.50 (6)	C24—C23—H23B	109.2
O10—Dy1—O1	90.94 (6)	C17—C23—H23B	109.2
O4—Dy1—O1	72.16 (7)	H23A—C23—H23B	107.9
O2—Dy1—O1	52.47 (6)	O7—C24—O8	120.1 (2)
O7—Dy1—O1	146.87 (6)	O7—C24—C23	120.6 (2)
O1W—Dy1—N1	81.08 (6)	O8—C24—C23	119.3 (2)
O5—Dy1—N1	130.28 (6)	O7—C24—Dy1	57.19 (12)
O10—Dy1—N1	154.16 (6)	O8—C24—Dy1	63.46 (12)
O4—Dy1—N1	76.27 (6)	C23—C24—Dy1	171.76 (19)
O2—Dy1—N1	74.17 (6)	C30—C25—C26	118.1 (2)
O7—Dy1—N1	75.04 (6)	C30—C25—C31	120.0 (2)
O1—Dy1—N1	86.87 (6)	C26—C25—C31	121.8 (3)
O1W—Dy1—O8	73.66 (6)	C27—C26—C25	120.9 (3)
O5—Dy1—O8	75.63 (5)	C27—C26—H26A	119.5
O10—Dy1—O8	65.72 (5)	C25—C26—H26A	119.5
O4—Dy1—O8	102.81 (6)	C28—C27—C26	119.8 (2)
O2—Dy1—O8	139.32 (5)	C28—C27—H27A	120.1
O7—Dy1—O8	51.83 (5)	C26—C27—H27A	120.1
O1—Dy1—O8	147.02 (5)	O12—C28—C27	122.4 (2)
N1—Dy1—O8	124.37 (6)	O12—C28—C29	117.6 (2)
O1W—Dy1—C16	163.52 (7)	C27—C28—C29	120.0 (2)
O5—Dy1—C16	27.39 (6)	C30—C29—C28	119.7 (3)
O10—Dy1—C16	100.39 (6)	C30—C29—H29A	120.1
O4—Dy1—C16	26.95 (6)	C28—C29—H29A	120.1
O2—Dy1—C16	121.84 (7)	C25—C30—C29	121.4 (2)
O7—Dy1—C16	87.57 (7)	C25—C30—H30A	119.3
O1—Dy1—C16	69.43 (7)	C29—C30—H30A	119.3
N1—Dy1—C16	102.89 (7)	C32—C31—C25	114.8 (2)
O8—Dy1—C16	91.27 (6)	C32—C31—H31A	108.6
O1W—Dy1—C8	100.85 (7)	C25—C31—H31A	108.6
O5—Dy1—C8	100.38 (7)	C32—C31—H31B	108.6
O10—Dy1—C8	89.57 (6)	C25—C31—H31B	108.6
O4—Dy1—C8	93.85 (7)	H31A—C31—H31B	107.5
O2—Dy1—C8	26.23 (7)	O11—C32—O10	119.3 (2)
O7—Dy1—C8	152.05 (6)	O11—C32—C31	122.9 (2)
O1—Dy1—C8	26.43 (6)	O10—C32—C31	117.8 (2)
N1—Dy1—C8	77.19 (7)	O11—C32—Dy2	60.85 (12)
O8—Dy1—C8	155.18 (6)	O10—C32—Dy2	59.63 (12)
C16—Dy1—C8	95.63 (7)	C31—C32—Dy2	168.86 (18)
O1W—Dy1—C24	72.21 (7)	C38—C33—C34	117.6 (2)
O5—Dy1—C24	87.01 (7)	C38—C33—C39	121.5 (2)
O10—Dy1—C24	91.77 (6)	C34—C33—C39	121.0 (2)
O4—Dy1—C24	91.16 (7)	C35—C34—C33	121.7 (2)
O2—Dy1—C24	146.80 (6)	C35—C34—H34A	119.2
O7—Dy1—C24	25.48 (6)	C33—C34—H34A	119.2
O1—Dy1—C24	160.73 (7)	C34—C35—C36	120.0 (2)
N1—Dy1—C24	98.72 (7)	C34—C35—H35A	120.0
O8—Dy1—C24	26.50 (6)	C36—C35—H35A	120.0
C16—Dy1—C24	91.33 (7)	O15—C36—C37	122.9 (2)
C8—Dy1—C24	172.56 (7)	O15—C36—C35	117.6 (2)
O8—Dy2—O14	128.78 (5)	C37—C36—C35	119.5 (2)
O8—Dy2—O2W	78.63 (6)	C36—C37—C38	119.9 (2)
O14—Dy2—O2W	143.23 (6)	C36—C37—H37A	120.1
O8—Dy2—O13	75.24 (5)	C38—C37—H37A	120.1
O14—Dy2—O13	54.16 (5)	C33—C38—C37	121.4 (2)
O2W—Dy2—O13	147.93 (6)	C33—C38—H38A	119.3
O8—Dy2—O16	80.48 (6)	C37—C38—H38A	119.3
O14—Dy2—O16	127.09 (6)	C40—C39—C33	115.6 (2)
O2W—Dy2—O16	75.56 (6)	C40—C39—H39A	108.4
O13—Dy2—O16	117.35 (6)	C33—C39—H39A	108.4
O8—Dy2—O17	98.92 (6)	C40—C39—H39B	108.4
O14—Dy2—O17	77.53 (6)	C33—C39—H39B	108.4
O2W—Dy2—O17	127.28 (6)	H39A—C39—H39B	107.4
O13—Dy2—O17	75.51 (6)	O14—C40—O13	119.3 (2)
O16—Dy2—O17	52.51 (6)	O14—C40—C39	118.9 (2)
O8—Dy2—O10	67.01 (5)	O13—C40—C39	121.8 (2)
O14—Dy2—O10	91.14 (6)	O14—C40—Dy2	59.17 (12)
O2W—Dy2—O10	77.11 (6)	O13—C40—Dy2	60.15 (12)
O13—Dy2—O10	75.78 (5)	C39—C40—Dy2	176.88 (17)
O16—Dy2—O10	140.85 (5)	C46—C41—C42	117.4 (2)
O17—Dy2—O10	150.47 (5)	C46—C41—C47	120.6 (3)
O8—Dy2—O11	115.47 (5)	C42—C41—C47	122.0 (2)
O14—Dy2—O11	72.89 (6)	C43—C42—C41	121.8 (3)
O2W—Dy2—O11	72.74 (6)	C43—C42—H42A	119.1
O13—Dy2—O11	102.26 (6)	C41—C42—H42A	119.1
O16—Dy2—O11	140.17 (6)	C42—C43—C44	119.8 (3)
O17—Dy2—O11	144.01 (6)	C42—C43—H43A	120.1
O10—Dy2—O11	50.94 (5)	C44—C43—H43A	120.1
O8—Dy2—N3	153.98 (6)	O18—C44—C43	117.7 (3)
O14—Dy2—N3	76.04 (6)	O18—C44—C45	122.7 (3)
O2W—Dy2—N3	83.47 (6)	C43—C44—C45	119.6 (3)
O13—Dy2—N3	126.95 (6)	C44—C45—C46	120.0 (3)
O16—Dy2—N3	76.84 (6)	C44—C45—H45A	120.0
O17—Dy2—N3	77.16 (6)	C46—C45—H45A	120.0
O10—Dy2—N3	126.89 (5)	C41—C46—C45	121.4 (3)
O11—Dy2—N3	76.19 (6)	C41—C46—H46A	119.3
O8—Dy2—C40	102.16 (6)	C45—C46—H46A	119.3
O14—Dy2—C40	27.02 (6)	C48—C47—C41	111.7 (2)
O2W—Dy2—C40	158.16 (7)	C48—C47—H47A	109.3
O13—Dy2—C40	27.15 (6)	C41—C47—H47A	109.3
O16—Dy2—C40	126.26 (6)	C48—C47—H47B	109.3
O17—Dy2—C40	74.43 (6)	C41—C47—H47B	109.3
O10—Dy2—C40	83.12 (6)	H47A—C47—H47B	107.9
O11—Dy2—C40	87.66 (6)	O16—C48—O17	119.2 (2)
N3—Dy2—C40	101.47 (6)	O16—C48—C47	120.1 (2)
O8—Dy2—C48	91.51 (6)	O17—C48—C47	120.8 (2)
O14—Dy2—C48	101.99 (7)	O16—C48—Dy2	58.01 (13)
O2W—Dy2—C48	100.92 (7)	O17—C48—Dy2	61.67 (13)
O13—Dy2—C48	98.06 (7)	C47—C48—Dy2	171.73 (16)
O16—Dy2—C48	26.14 (7)	N1—C49—C50	123.6 (2)
O17—Dy2—C48	26.52 (7)	N1—C49—H49A	118.2
O10—Dy2—C48	158.48 (6)	C50—C49—H49A	118.2
O11—Dy2—C48	149.50 (6)	C51—C50—C49	119.4 (2)
N3—Dy2—C48	73.42 (6)	C51—C50—H50A	120.3
C40—Dy2—C48	100.87 (7)	C49—C50—H50A	120.3
O8—Dy2—C32	90.63 (6)	C50—C51—C52	117.1 (2)
O14—Dy2—C32	83.91 (7)	C50—C51—C56	121.1 (2)
O2W—Dy2—C32	70.31 (7)	C52—C51—C56	121.8 (2)
O13—Dy2—C32	91.58 (6)	C53—C52—C51	119.8 (2)
O16—Dy2—C32	145.79 (6)	C53—C52—H52A	120.1
O17—Dy2—C32	161.26 (6)	C51—C52—H52A	120.1
O10—Dy2—C32	25.89 (5)	N1—C53—C52	123.6 (2)
O11—Dy2—C32	25.37 (6)	N1—C53—H53A	118.2
N3—Dy2—C32	101.00 (6)	C52—C53—H53A	118.2
C40—Dy2—C32	87.85 (7)	N2—C54—C55	124.5 (3)
C48—Dy2—C32	170.36 (7)	N2—C54—H54A	117.8
C49—N1—C53	116.5 (2)	C55—C54—H54A	117.8
C49—N1—Dy1	124.22 (16)	C56—C55—C54	119.0 (3)
C53—N1—Dy1	118.95 (16)	C56—C55—H55A	120.5
C58—N2—C54	115.6 (3)	C54—C55—H55A	120.5
C59—N3—C63	116.4 (2)	C55—C56—C57	116.9 (3)
C59—N3—Dy2	121.40 (16)	C55—C56—C51	121.0 (3)
C63—N3—Dy2	121.92 (16)	C57—C56—C51	121.9 (3)
C64—N4—C68	116.3 (3)	C56—C57—C58	119.1 (3)
C2—C1—C6	117.8 (2)	C56—C57—H57A	120.4
C2—C1—C7	122.6 (2)	C58—C57—H57A	120.4
C6—C1—C7	119.6 (2)	N2—C58—C57	124.9 (3)
C1—C2—C3	121.6 (2)	N2—C58—H58A	117.6
C1—C2—H2A	119.2	C57—C58—H58A	117.6
C3—C2—H2A	119.2	N3—C59—C60	123.3 (2)
C4—C3—C2	119.8 (2)	N3—C59—H59A	118.3
C4—C3—H3A	120.1	C60—C59—H59A	118.3
C2—C3—H3A	120.1	C61—C60—C59	120.3 (2)
O3—C4—C5	121.8 (2)	C61—C60—H60A	119.8
O3—C4—C3	118.5 (2)	C59—C60—H60A	119.8
C5—C4—C3	119.6 (2)	C60—C61—C62	116.2 (2)
C4—C5—C6	120.1 (3)	C60—C61—C66	121.0 (2)
C4—C5—H5A	119.9	C62—C61—C66	122.8 (2)
C6—C5—H5A	119.9	C63—C62—C61	120.1 (2)
C5—C6—C1	121.1 (2)	C63—C62—H62A	119.9
C5—C6—H6A	119.5	C61—C62—H62A	119.9
C1—C6—H6A	119.5	N3—C63—C62	123.5 (2)
C8—C7—C1	111.2 (2)	N3—C63—H63A	118.2
C8—C7—H7A	109.4	C62—C63—H63A	118.2
C1—C7—H7A	109.4	N4—C64—C65	124.0 (3)
C8—C7—H7B	109.4	N4—C64—H64A	118.0
C1—C7—H7B	109.4	C65—C64—H64A	118.0
H7A—C7—H7B	108.0	C64—C65—C66	119.4 (3)
O2—C8—O1	119.5 (2)	C64—C65—H65A	120.3
O2—C8—C7	119.6 (2)	C66—C65—H65A	120.3
O1—C8—C7	120.9 (2)	C67—C66—C65	116.8 (3)
O2—C8—Dy1	57.54 (13)	C67—C66—C61	121.1 (3)
O1—C8—Dy1	62.64 (13)	C65—C66—C61	122.0 (3)
C7—C8—Dy1	169.54 (16)	C66—C67—C68	119.3 (3)
C10—C9—C14	117.2 (2)	C66—C67—H67A	120.3
C10—C9—C15	122.2 (3)	C68—C67—H67A	120.3
C14—C9—C15	120.6 (3)	N4—C68—C67	124.1 (4)
C9—C10—C11	122.2 (3)	N4—C68—H68A	118.0
C9—C10—H10A	118.9	C67—C68—H68A	118.0
C11—C10—H10A	118.9	Dy1—O1W—H1WA	124 (3)
C12—C11—C10	119.3 (3)	Dy1—O1W—H1WB	133 (3)
C12—C11—H11A	120.4	H1WA—O1W—H1WB	103 (2)
C10—C11—H11A	120.4	C8—O1—Dy1	90.93 (15)
C11—C12—C13	119.6 (2)	Dy2—O2W—H2WA	122 (3)
C11—C12—O6	122.6 (2)	Dy2—O2W—H2WB	135 (3)
C13—C12—O6	117.8 (2)	H2WA—O2W—H2WB	103 (2)
C14—C13—C12	120.5 (3)	C8—O2—Dy1	96.23 (15)
C14—C13—H13A	119.8	C4—O3—H3B	109.5
C12—C13—H13A	119.8	H3WB—O3W—H3WA	103 (2)
C13—C14—C9	121.3 (3)	C16—O4—Dy1	92.51 (14)
C13—C14—H14A	119.4	C16—O5—Dy1	92.47 (13)
C9—C14—H14A	119.4	C12—O6—H6B	109.5
C9—C15—C16	115.7 (2)	C24—O7—Dy1	97.33 (14)
C9—C15—H15A	108.3	C24—O8—Dy2	153.71 (16)
C16—C15—H15A	108.3	C24—O8—Dy1	90.05 (14)
C9—C15—H15B	108.3	Dy2—O8—Dy1	113.91 (6)
C16—C15—H15B	108.3	C20—O9—H9A	109.5
H15A—C15—H15B	107.4	C32—O10—Dy1	142.46 (15)
O4—C16—O5	120.0 (2)	C32—O10—Dy2	94.48 (13)
O4—C16—C15	119.6 (2)	Dy1—O10—Dy2	113.35 (6)
O5—C16—C15	120.3 (2)	C32—O11—Dy2	93.79 (13)
O4—C16—Dy1	60.53 (12)	C28—O12—H12A	109.5
O5—C16—Dy1	60.14 (12)	C40—O13—Dy2	92.70 (13)
C15—C16—Dy1	169.7 (2)	C40—O14—Dy2	93.81 (14)
C22—C17—C18	117.6 (2)	C36—O15—H15C	109.5
C22—C17—C23	121.6 (3)	C48—O16—Dy2	95.85 (15)
C18—C17—C23	120.7 (3)	C48—O17—Dy2	91.81 (15)
C19—C18—C17	121.3 (2)	C44—O18—H18B	109.5
C19—C18—H18A	119.3		
			
O1W—Dy1—N1—C49	51.33 (19)	O2W—Dy2—C48—O17	174.16 (14)
O5—Dy1—N1—C49	−144.87 (18)	O13—Dy2—C48—O17	−31.82 (15)
O10—Dy1—N1—C49	8.8 (3)	O16—Dy2—C48—O17	−171.7 (2)
O4—Dy1—N1—C49	−149.3 (2)	O10—Dy2—C48—O17	−103.3 (2)
O2—Dy1—N1—C49	−25.12 (18)	O11—Dy2—C48—O17	99.69 (18)
O7—Dy1—N1—C49	131.1 (2)	N3—Dy2—C48—O17	94.43 (15)
O1—Dy1—N1—C49	−76.90 (19)	C40—Dy2—C48—O17	−4.43 (16)
O8—Dy1—N1—C49	114.43 (19)	C53—N1—C49—C50	1.4 (4)
C16—Dy1—N1—C49	−144.97 (19)	Dy1—N1—C49—C50	−172.38 (19)
C8—Dy1—N1—C49	−52.03 (19)	N1—C49—C50—C51	0.6 (4)
C24—Dy1—N1—C49	121.65 (19)	C49—C50—C51—C52	−2.2 (4)
O1W—Dy1—N1—C53	−122.30 (19)	C49—C50—C51—C56	176.1 (2)
O5—Dy1—N1—C53	41.5 (2)	C50—C51—C52—C53	1.9 (4)
O10—Dy1—N1—C53	−164.83 (16)	C56—C51—C52—C53	−176.3 (3)
O4—Dy1—N1—C53	37.08 (18)	C49—N1—C53—C52	−1.7 (4)
O2—Dy1—N1—C53	161.25 (19)	Dy1—N1—C53—C52	172.4 (2)
O7—Dy1—N1—C53	−42.50 (18)	C51—C52—C53—N1	0.0 (4)
O1—Dy1—N1—C53	109.47 (19)	C58—N2—C54—C55	1.3 (5)
O8—Dy1—N1—C53	−59.2 (2)	N2—C54—C55—C56	−0.2 (5)
C16—Dy1—N1—C53	41.4 (2)	C54—C55—C56—C57	−1.4 (5)
C8—Dy1—N1—C53	134.34 (19)	C54—C55—C56—C51	175.4 (3)
C24—Dy1—N1—C53	−51.98 (19)	C50—C51—C56—C55	−149.2 (3)
O8—Dy2—N3—C59	0.4 (3)	C52—C51—C56—C55	29.0 (4)
O14—Dy2—N3—C59	164.50 (19)	C50—C51—C56—C57	27.5 (4)
O2W—Dy2—N3—C59	−46.27 (18)	C52—C51—C56—C57	−154.3 (3)
O13—Dy2—N3—C59	144.87 (17)	C55—C56—C57—C58	1.8 (5)
O16—Dy2—N3—C59	30.37 (18)	C51—C56—C57—C58	−175.0 (3)
O17—Dy2—N3—C59	84.38 (19)	C54—N2—C58—C57	−0.8 (5)
O10—Dy2—N3—C59	−114.75 (18)	C56—C57—C58—N2	−0.7 (6)
O11—Dy2—N3—C59	−120.04 (19)	C63—N3—C59—C60	−2.2 (4)
C40—Dy2—N3—C59	155.28 (18)	Dy2—N3—C59—C60	171.7 (2)
C48—Dy2—N3—C59	57.21 (18)	N3—C59—C60—C61	−1.2 (4)
C32—Dy2—N3—C59	−114.69 (18)	C59—C60—C61—C62	3.6 (4)
O8—Dy2—N3—C63	173.99 (16)	C59—C60—C61—C66	−173.6 (2)
O14—Dy2—N3—C63	−21.87 (18)	C60—C61—C62—C63	−2.6 (4)
O2W—Dy2—N3—C63	127.36 (19)	C66—C61—C62—C63	174.5 (2)
O13—Dy2—N3—C63	−41.5 (2)	C59—N3—C63—C62	3.3 (4)
O16—Dy2—N3—C63	−156.00 (19)	Dy2—N3—C63—C62	−170.6 (2)
O17—Dy2—N3—C63	−101.99 (19)	C61—C62—C63—N3	−0.9 (4)
O10—Dy2—N3—C63	58.9 (2)	C68—N4—C64—C65	0.1 (5)
O11—Dy2—N3—C63	53.59 (18)	N4—C64—C65—C66	1.8 (5)
C40—Dy2—N3—C63	−31.09 (19)	C64—C65—C66—C67	−2.1 (4)
C48—Dy2—N3—C63	−129.2 (2)	C64—C65—C66—C61	174.3 (3)
C32—Dy2—N3—C63	58.94 (19)	C60—C61—C66—C67	159.6 (3)
C6—C1—C2—C3	−1.3 (4)	C62—C61—C66—C67	−17.5 (4)
C7—C1—C2—C3	−179.1 (3)	C60—C61—C66—C65	−16.7 (4)
C1—C2—C3—C4	0.4 (4)	C62—C61—C66—C65	166.3 (3)
C2—C3—C4—O3	−179.5 (3)	C65—C66—C67—C68	0.7 (4)
C2—C3—C4—C5	1.3 (4)	C61—C66—C67—C68	−175.8 (3)
O3—C4—C5—C6	178.8 (3)	C64—N4—C68—C67	−1.6 (5)
C3—C4—C5—C6	−2.0 (4)	C66—C67—C68—N4	1.3 (5)
C4—C5—C6—C1	1.0 (4)	O2—C8—O1—Dy1	9.2 (2)
C2—C1—C6—C5	0.6 (4)	C7—C8—O1—Dy1	−168.7 (2)
C7—C1—C6—C5	178.4 (3)	O1W—Dy1—O1—C8	−9.26 (17)
C2—C1—C7—C8	−48.1 (3)	O5—Dy1—O1—C8	−159.82 (15)
C6—C1—C7—C8	134.2 (3)	O10—Dy1—O1—C8	−87.13 (14)
C1—C7—C8—O2	−80.7 (3)	O4—Dy1—O1—C8	143.69 (15)
C1—C7—C8—O1	97.2 (3)	O2—Dy1—O1—C8	−5.28 (13)
C1—C7—C8—Dy1	−9.7 (12)	O7—Dy1—O1—C8	123.28 (15)
O1W—Dy1—C8—O2	1.98 (14)	N1—Dy1—O1—C8	67.11 (14)
O5—Dy1—C8—O2	−150.65 (13)	O8—Dy1—O1—C8	−130.22 (14)
O10—Dy1—C8—O2	−77.51 (14)	C16—Dy1—O1—C8	172.12 (16)
O4—Dy1—C8—O2	155.10 (14)	C24—Dy1—O1—C8	174.79 (18)
O7—Dy1—C8—O2	86.7 (2)	O1—C8—O2—Dy1	−9.7 (2)
O1—Dy1—C8—O2	−170.5 (2)	C7—C8—O2—Dy1	168.26 (18)
N1—Dy1—C8—O2	80.12 (14)	O1W—Dy1—O2—C8	−177.98 (15)
O8—Dy1—C8—O2	−72.5 (2)	O5—Dy1—O2—C8	34.96 (16)
C16—Dy1—C8—O2	−177.91 (14)	O10—Dy1—O2—C8	101.05 (14)
O1W—Dy1—C8—O1	172.48 (14)	O4—Dy1—O2—C8	−28.24 (15)
O5—Dy1—C8—O1	19.85 (15)	O7—Dy1—O2—C8	−131.57 (14)
O10—Dy1—C8—O1	92.99 (14)	O1—Dy1—O2—C8	5.31 (13)
O4—Dy1—C8—O1	−34.40 (14)	N1—Dy1—O2—C8	−93.10 (14)
O2—Dy1—C8—O1	170.5 (2)	O8—Dy1—O2—C8	142.12 (13)
O7—Dy1—C8—O1	−102.84 (19)	C16—Dy1—O2—C8	2.45 (16)
N1—Dy1—C8—O1	−109.38 (15)	C24—Dy1—O2—C8	−174.73 (13)
O8—Dy1—C8—O1	98.03 (19)	O5—C16—O4—Dy1	9.2 (3)
C16—Dy1—C8—O1	−7.41 (15)	C15—C16—O4—Dy1	−168.1 (2)
O1W—Dy1—C8—C7	−75.1 (10)	O1W—Dy1—O4—C16	−146.09 (15)
O5—Dy1—C8—C7	132.3 (10)	O5—Dy1—O4—C16	−5.17 (14)
O10—Dy1—C8—C7	−154.5 (10)	O10—Dy1—O4—C16	2.47 (18)
O4—Dy1—C8—C7	78.1 (10)	O2—Dy1—O4—C16	106.96 (16)
O2—Dy1—C8—C7	−77.0 (10)	O7—Dy1—O4—C16	−111.75 (16)
O7—Dy1—C8—C7	9.6 (11)	O1—Dy1—O4—C16	79.54 (16)
O1—Dy1—C8—C7	112.5 (11)	N1—Dy1—O4—C16	170.67 (17)
N1—Dy1—C8—C7	3.1 (10)	O8—Dy1—O4—C16	−66.61 (16)
O8—Dy1—C8—C7	−149.5 (10)	C8—Dy1—O4—C16	94.86 (16)
C16—Dy1—C8—C7	105.1 (10)	C24—Dy1—O4—C16	−90.64 (16)
C14—C9—C10—C11	1.0 (4)	O4—C16—O5—Dy1	−9.2 (3)
C15—C9—C10—C11	−179.1 (3)	C15—C16—O5—Dy1	168.1 (2)
C9—C10—C11—C12	−0.6 (5)	O1W—Dy1—O5—C16	150.68 (15)
C10—C11—C12—C13	0.1 (4)	O10—Dy1—O5—C16	−168.57 (16)
C10—C11—C12—O6	178.8 (3)	O4—Dy1—O5—C16	5.10 (14)
C11—C12—C13—C14	0.1 (4)	O2—Dy1—O5—C16	−97.06 (15)
O6—C12—C13—C14	−178.7 (3)	O7—Dy1—O5—C16	74.53 (15)
C12—C13—C14—C9	0.3 (4)	O1—Dy1—O5—C16	−73.16 (15)
C10—C9—C14—C13	−0.8 (4)	N1—Dy1—O5—C16	−0.20 (18)
C15—C9—C14—C13	179.3 (3)	O8—Dy1—O5—C16	122.95 (15)
C10—C9—C15—C16	−98.1 (3)	C8—Dy1—O5—C16	−82.14 (15)
C14—C9—C15—C16	81.8 (3)	C24—Dy1—O5—C16	98.70 (15)
C9—C15—C16—O4	172.1 (3)	O8—C24—O7—Dy1	−8.6 (3)
C9—C15—C16—O5	−5.2 (4)	C23—C24—O7—Dy1	170.7 (2)
C9—C15—C16—Dy1	84.4 (10)	O1W—Dy1—O7—C24	−73.89 (16)
O1W—Dy1—C16—O4	93.0 (3)	O5—Dy1—O7—C24	71.88 (16)
O5—Dy1—C16—O4	170.9 (3)	O10—Dy1—O7—C24	−2.21 (17)
O10—Dy1—C16—O4	−178.00 (15)	O4—Dy1—O7—C24	123.15 (16)
O2—Dy1—C16—O4	−88.50 (16)	O2—Dy1—O7—C24	−119.47 (16)
O7—Dy1—C16—O4	64.75 (16)	O1—Dy1—O7—C24	143.10 (15)
O1—Dy1—C16—O4	−90.92 (16)	N1—Dy1—O7—C24	−157.75 (17)
N1—Dy1—C16—O4	−9.30 (17)	O8—Dy1—O7—C24	4.69 (14)
O8—Dy1—C16—O4	116.46 (15)	C16—Dy1—O7—C24	98.22 (16)
C8—Dy1—C16—O4	−87.41 (16)	C8—Dy1—O7—C24	−164.34 (16)
C24—Dy1—C16—O4	89.96 (16)	O7—C24—O8—Dy2	164.5 (2)
O1W—Dy1—C16—O5	−77.9 (3)	C23—C24—O8—Dy2	−14.7 (5)
O10—Dy1—C16—O5	11.14 (15)	Dy1—C24—O8—Dy2	156.5 (3)
O4—Dy1—C16—O5	−170.9 (3)	O7—C24—O8—Dy1	8.1 (2)
O2—Dy1—C16—O5	100.64 (15)	C23—C24—O8—Dy1	−171.2 (2)
O7—Dy1—C16—O5	−106.11 (15)	O14—Dy2—O8—C24	−82.2 (4)
O1—Dy1—C16—O5	98.22 (15)	O2W—Dy2—O8—C24	125.3 (4)
N1—Dy1—C16—O5	179.85 (14)	O13—Dy2—O8—C24	−73.5 (3)
O8—Dy1—C16—O5	−54.40 (14)	O16—Dy2—O8—C24	48.3 (3)
C8—Dy1—C16—O5	101.73 (15)	O17—Dy2—O8—C24	−1.1 (4)
C24—Dy1—C16—O5	−80.90 (15)	O10—Dy2—O8—C24	−154.0 (4)
O1W—Dy1—C16—C15	−173.4 (8)	O11—Dy2—O8—C24	−170.2 (3)
O5—Dy1—C16—C15	−95.6 (10)	N3—Dy2—O8—C24	77.9 (4)
O10—Dy1—C16—C15	−84.4 (10)	C40—Dy2—O8—C24	−77.0 (4)
O4—Dy1—C16—C15	93.6 (10)	C48—Dy2—O8—C24	24.5 (3)
O2—Dy1—C16—C15	5.1 (10)	C32—Dy2—O8—C24	−164.9 (3)
O7—Dy1—C16—C15	158.3 (10)	O14—Dy2—O8—Dy1	71.87 (9)
O1—Dy1—C16—C15	2.7 (9)	O2W—Dy2—O8—Dy1	−80.57 (7)
N1—Dy1—C16—C15	84.3 (10)	O13—Dy2—O8—Dy1	80.66 (7)
O8—Dy1—C16—C15	−150.0 (9)	O16—Dy2—O8—Dy1	−157.61 (7)
C8—Dy1—C16—C15	6.2 (10)	O17—Dy2—O8—Dy1	153.00 (6)
C24—Dy1—C16—C15	−176.5 (9)	O10—Dy2—O8—Dy1	0.11 (5)
C22—C17—C18—C19	0.2 (4)	O11—Dy2—O8—Dy1	−16.10 (9)
C23—C17—C18—C19	179.2 (2)	N3—Dy2—O8—Dy1	−128.02 (12)
C17—C18—C19—C20	0.2 (4)	C40—Dy2—O8—Dy1	77.13 (7)
C18—C19—C20—O9	179.7 (2)	C48—Dy2—O8—Dy1	178.59 (7)
C18—C19—C20—C21	−0.6 (4)	C32—Dy2—O8—Dy1	−10.81 (7)
O9—C20—C21—C22	−179.6 (2)	O1W—Dy1—O8—C24	82.80 (14)
C19—C20—C21—C22	0.7 (4)	O5—Dy1—O8—C24	−113.14 (14)
C20—C21—C22—C17	−0.3 (4)	O10—Dy1—O8—C24	168.74 (15)
C18—C17—C22—C21	−0.1 (4)	O4—Dy1—O8—C24	−65.84 (14)
C23—C17—C22—C21	−179.2 (2)	O2—Dy1—O8—C24	122.93 (14)
C22—C17—C23—C24	99.0 (3)	O7—Dy1—O8—C24	−4.53 (13)
C18—C17—C23—C24	−80.1 (3)	O1—Dy1—O8—C24	−142.72 (14)
C17—C23—C24—O7	−45.9 (4)	N1—Dy1—O8—C24	16.15 (16)
C17—C23—C24—O8	133.3 (3)	C16—Dy1—O8—C24	−90.43 (14)
O1W—Dy1—C24—O7	99.37 (16)	C8—Dy1—O8—C24	163.21 (17)
O5—Dy1—C24—O7	−108.59 (16)	O1W—Dy1—O8—Dy2	−86.05 (7)
O10—Dy1—C24—O7	178.03 (16)	O5—Dy1—O8—Dy2	78.00 (7)
O4—Dy1—C24—O7	−54.57 (16)	O10—Dy1—O8—Dy2	−0.12 (5)
O2—Dy1—C24—O7	96.07 (18)	O4—Dy1—O8—Dy2	125.31 (7)
O1—Dy1—C24—O7	−84.0 (2)	O2—Dy1—O8—Dy2	−45.92 (11)
N1—Dy1—C24—O7	21.72 (16)	O7—Dy1—O8—Dy2	−173.38 (10)
O8—Dy1—C24—O7	−171.7 (2)	O1—Dy1—O8—Dy2	48.43 (13)
C16—Dy1—C24—O7	−81.53 (16)	N1—Dy1—O8—Dy2	−152.70 (7)
O1W—Dy1—C24—O8	−88.92 (14)	C16—Dy1—O8—Dy2	100.72 (7)
O5—Dy1—C24—O8	63.12 (13)	C8—Dy1—O8—Dy2	−5.64 (18)
O10—Dy1—C24—O8	−10.26 (14)	C24—Dy1—O8—Dy2	−168.85 (17)
O4—Dy1—C24—O8	117.14 (13)	O11—C32—O10—Dy1	−151.33 (18)
O2—Dy1—C24—O8	−92.22 (16)	C31—C32—O10—Dy1	28.6 (4)
O7—Dy1—C24—O8	171.7 (2)	Dy2—C32—O10—Dy1	−139.0 (2)
O1—Dy1—C24—O8	87.7 (2)	O11—C32—O10—Dy2	−12.3 (2)
N1—Dy1—C24—O8	−166.57 (13)	C31—C32—O10—Dy2	167.6 (2)
C16—Dy1—C24—O8	90.18 (14)	O1W—Dy1—O10—C32	−148.8 (2)
C30—C25—C26—C27	−0.4 (4)	O5—Dy1—O10—C32	53.3 (2)
C31—C25—C26—C27	177.9 (2)	O4—Dy1—O10—C32	46.8 (3)
C25—C26—C27—C28	−0.7 (4)	O2—Dy1—O10—C32	−73.4 (2)
C26—C27—C28—O12	−179.1 (2)	O7—Dy1—O10—C32	140.6 (2)
C26—C27—C28—C29	1.3 (4)	O1—Dy1—O10—C32	−21.3 (2)
O12—C28—C29—C30	179.6 (2)	N1—Dy1—O10—C32	−106.0 (3)
C27—C28—C29—C30	−0.7 (4)	O8—Dy1—O10—C32	134.7 (3)
C26—C25—C30—C29	1.0 (4)	C16—Dy1—O10—C32	48.0 (2)
C31—C25—C30—C29	−177.4 (2)	C8—Dy1—O10—C32	−47.7 (2)
C28—C29—C30—C25	−0.5 (4)	C24—Dy1—O10—C32	139.7 (2)
C30—C25—C31—C32	75.4 (3)	O1W—Dy1—O10—Dy2	76.68 (7)
C26—C25—C31—C32	−102.9 (3)	O5—Dy1—O10—Dy2	−81.24 (7)
C25—C31—C32—O11	12.1 (4)	O4—Dy1—O10—Dy2	−87.70 (9)
C25—C31—C32—O10	−167.9 (2)	O2—Dy1—O10—Dy2	152.08 (7)
C25—C31—C32—Dy2	−94.3 (8)	O7—Dy1—O10—Dy2	6.06 (9)
O8—Dy2—C32—O11	−168.79 (14)	O1—Dy1—O10—Dy2	−155.81 (7)
O14—Dy2—C32—O11	62.26 (14)	N1—Dy1—O10—Dy2	119.41 (13)
O2W—Dy2—C32—O11	−91.10 (14)	O8—Dy1—O10—Dy2	0.11 (5)
O13—Dy2—C32—O11	115.96 (14)	C16—Dy1—O10—Dy2	−86.56 (8)
O16—Dy2—C32—O11	−94.94 (16)	C8—Dy1—O10—Dy2	177.79 (8)
O17—Dy2—C32—O11	70.2 (2)	C24—Dy1—O10—Dy2	5.11 (7)
O10—Dy2—C32—O11	167.7 (2)	O8—Dy2—O10—C32	−154.30 (14)
N3—Dy2—C32—O11	−12.21 (15)	O14—Dy2—O10—C32	73.49 (13)
C40—Dy2—C32—O11	89.06 (14)	O2W—Dy2—O10—C32	−71.33 (13)
O8—Dy2—C32—O10	23.53 (13)	O13—Dy2—O10—C32	125.95 (14)
O14—Dy2—C32—O10	−105.42 (13)	O16—Dy2—O10—C32	−117.97 (14)
O2W—Dy2—C32—O10	101.22 (14)	O17—Dy2—O10—C32	139.73 (15)
O13—Dy2—C32—O10	−51.72 (13)	O11—Dy2—O10—C32	6.76 (12)
O16—Dy2—C32—O10	97.39 (15)	N3—Dy2—O10—C32	0.14 (15)
O17—Dy2—C32—O10	−97.5 (2)	C40—Dy2—O10—C32	99.34 (14)
O11—Dy2—C32—O10	−167.7 (2)	C48—Dy2—O10—C32	−158.43 (18)
N3—Dy2—C32—O10	−179.88 (12)	O8—Dy2—O10—Dy1	−0.12 (5)
C40—Dy2—C32—O10	−78.61 (13)	O14—Dy2—O10—Dy1	−132.33 (7)
O8—Dy2—C32—C31	−56.1 (8)	O2W—Dy2—O10—Dy1	82.85 (7)
O14—Dy2—C32—C31	175.0 (8)	O13—Dy2—O10—Dy1	−79.87 (7)
O2W—Dy2—C32—C31	21.6 (8)	O16—Dy2—O10—Dy1	36.21 (11)
O13—Dy2—C32—C31	−131.3 (8)	O17—Dy2—O10—Dy1	−66.09 (14)
O16—Dy2—C32—C31	17.8 (9)	O11—Dy2—O10—Dy1	160.94 (10)
O17—Dy2—C32—C31	−177.1 (7)	N3—Dy2—O10—Dy1	154.32 (7)
O10—Dy2—C32—C31	−79.6 (8)	C40—Dy2—O10—Dy1	−106.48 (7)
O11—Dy2—C32—C31	112.7 (9)	C48—Dy2—O10—Dy1	−4.2 (2)
N3—Dy2—C32—C31	100.5 (8)	C32—Dy2—O10—Dy1	154.18 (16)
C40—Dy2—C32—C31	−158.2 (8)	O10—C32—O11—Dy2	12.2 (2)
C38—C33—C34—C35	−0.7 (4)	C31—C32—O11—Dy2	−167.7 (2)
C39—C33—C34—C35	179.1 (2)	O8—Dy2—O11—C32	12.44 (15)
C33—C34—C35—C36	0.5 (4)	O14—Dy2—O11—C32	−112.96 (14)
C34—C35—C36—O15	179.9 (2)	O2W—Dy2—O11—C32	80.31 (14)
C34—C35—C36—C37	−0.1 (4)	O13—Dy2—O11—C32	−66.89 (14)
O15—C36—C37—C38	−180.0 (2)	O16—Dy2—O11—C32	119.01 (14)
C35—C36—C37—C38	0.0 (4)	O17—Dy2—O11—C32	−149.04 (13)
C34—C33—C38—C37	0.6 (4)	O10—Dy2—O11—C32	−6.89 (13)
C39—C33—C38—C37	−179.2 (2)	N3—Dy2—O11—C32	167.66 (15)
C36—C37—C38—C33	−0.3 (4)	C40—Dy2—O11—C32	−89.93 (14)
C38—C33—C39—C40	−114.6 (3)	C48—Dy2—O11—C32	162.46 (15)
C34—C33—C39—C40	65.6 (3)	O14—C40—O13—Dy2	1.5 (2)
C33—C39—C40—O14	173.1 (2)	C39—C40—O13—Dy2	−177.2 (2)
C33—C39—C40—O13	−8.2 (4)	O8—Dy2—O13—C40	−172.42 (15)
O8—Dy2—C40—O14	−170.95 (14)	O14—Dy2—O13—C40	−0.87 (13)
O2W—Dy2—C40—O14	−81.2 (2)	O2W—Dy2—O13—C40	−135.97 (15)
O13—Dy2—C40—O14	−178.4 (2)	O16—Dy2—O13—C40	116.77 (14)
O16—Dy2—C40—O14	102.01 (15)	O17—Dy2—O13—C40	84.06 (14)
O17—Dy2—C40—O14	93.04 (15)	O10—Dy2—O13—C40	−102.90 (14)
O10—Dy2—C40—O14	−106.32 (15)	O11—Dy2—O13—C40	−58.98 (14)
O11—Dy2—C40—O14	−55.39 (14)	N3—Dy2—O13—C40	22.85 (17)
N3—Dy2—C40—O14	20.01 (15)	C48—Dy2—O13—C40	98.13 (15)
C48—Dy2—C40—O14	95.09 (15)	C32—Dy2—O13—C40	−82.19 (14)
C32—Dy2—C40—O14	−80.78 (15)	O13—C40—O14—Dy2	−1.5 (2)
O8—Dy2—C40—O13	7.49 (15)	C39—C40—O14—Dy2	177.20 (19)
O14—Dy2—C40—O13	178.4 (2)	O8—Dy2—O14—C40	11.37 (18)
O2W—Dy2—C40—O13	97.3 (2)	O2W—Dy2—O14—C40	142.11 (14)
O16—Dy2—C40—O13	−79.54 (15)	O13—Dy2—O14—C40	0.87 (13)
O17—Dy2—C40—O13	−88.51 (14)	O16—Dy2—O14—C40	−98.60 (15)
O10—Dy2—C40—O13	72.13 (14)	O17—Dy2—O14—C40	−80.13 (15)
O11—Dy2—C40—O13	123.06 (14)	O10—Dy2—O14—C40	72.36 (15)
N3—Dy2—C40—O13	−161.54 (14)	O11—Dy2—O14—C40	120.63 (15)
C48—Dy2—C40—O13	−86.46 (14)	N3—Dy2—O14—C40	−159.78 (16)
C32—Dy2—C40—O13	97.67 (14)	C48—Dy2—O14—C40	−90.47 (15)
C46—C41—C42—C43	−1.0 (4)	C32—Dy2—O14—C40	97.26 (15)
C47—C41—C42—C43	177.5 (2)	O17—C48—O16—Dy2	8.4 (2)
C41—C42—C43—C44	−0.7 (4)	C47—C48—O16—Dy2	−170.62 (19)
C42—C43—C44—O18	−178.6 (3)	O8—Dy2—O16—C48	−113.73 (14)
C42—C43—C44—C45	1.7 (4)	O14—Dy2—O16—C48	18.29 (16)
O18—C44—C45—C46	179.3 (3)	O2W—Dy2—O16—C48	165.66 (15)
C43—C44—C45—C46	−1.0 (4)	O13—Dy2—O16—C48	−45.91 (15)
C42—C41—C46—C45	1.7 (4)	O17—Dy2—O16—C48	−4.66 (13)
C47—C41—C46—C45	−176.8 (2)	O10—Dy2—O16—C48	−147.30 (13)
C44—C45—C46—C41	−0.7 (4)	O11—Dy2—O16—C48	127.60 (14)
C46—C41—C47—C48	107.5 (3)	N3—Dy2—O16—C48	79.13 (14)
C42—C41—C47—C48	−70.9 (3)	C40—Dy2—O16—C48	−15.57 (16)
C41—C47—C48—O16	58.5 (3)	C32—Dy2—O16—C48	169.39 (13)
C41—C47—C48—O17	−120.4 (3)	O16—C48—O17—Dy2	−8.1 (2)
O8—Dy2—C48—O16	64.58 (14)	C47—C48—O17—Dy2	170.9 (2)
O14—Dy2—C48—O16	−165.18 (13)	O8—Dy2—O17—C48	75.24 (15)
O2W—Dy2—C48—O16	−14.13 (14)	O14—Dy2—O17—C48	−156.84 (15)
O13—Dy2—C48—O16	139.89 (13)	O2W—Dy2—O17—C48	−7.21 (18)
O17—Dy2—C48—O16	171.7 (2)	O13—Dy2—O17—C48	147.37 (16)
O10—Dy2—C48—O16	68.4 (2)	O16—Dy2—O17—C48	4.59 (13)
O11—Dy2—C48—O16	−88.60 (19)	O10—Dy2—O17—C48	133.58 (14)
N3—Dy2—C48—O16	−93.87 (14)	O11—Dy2—O17—C48	−121.63 (15)
C40—Dy2—C48—O16	167.27 (14)	N3—Dy2—O17—C48	−78.55 (15)
O8—Dy2—C48—O17	−107.13 (15)	C40—Dy2—O17—C48	175.48 (16)
O14—Dy2—C48—O17	23.12 (15)	C32—Dy2—O17—C48	−164.95 (18)

### Hydrogen-bond geometry (Å, °)

**Table:**

*D*—H···*A*	*D*—H	H···*A*	*D*···*A*	*D*—H···*A*
O3—H3B···O12^i^	0.82	1.93	2.742 (3)	169
O6—H6B···O3W^ii^	0.82	1.86	2.644 (3)	160
O9—H9A···O17^iii^	0.82	1.85	2.670 (3)	173
O12—H12A···O11^iv^	0.82	1.94	2.748 (2)	168
O15—H15C···O6^v^	0.82	1.90	2.715 (3)	174
O18—H18B···O9^ii^	0.82	1.95	2.766 (3)	174
O2W—H2WA···O5	0.84 (4)	1.96 (2)	2.745 (2)	154 (4)
O2W—H2WB···N2^ii^	0.84 (2)	2.04 (2)	2.834 (3)	160 (4)
O3W—H3WB···O3	0.83 (4)	1.99 (2)	2.799 (3)	163 (4)
O1W—H1WA···O13	0.82 (4)	1.98 (2)	2.738 (2)	153 (4)
O1W—H1WB···N4^i^	0.84 (2)	1.96 (2)	2.781 (3)	167 (4)
O3W—H3WA···O1^vi^	0.84 (4)	1.94 (2)	2.775 (3)	178 (4)

Symmetry codes: (i) *x*, *y*+1, *z*; (ii) *x*, *y*−1, *z*; (iii) −*x*, −*y*+1, −*z*; (iv) −*x*, −*y*, −*z*+1; (v) *x*−1, *y*+1, *z*; (vi) −*x*+1, −*y*+1, −*z*+1.
